# Farmer and Consumer Responses to African Swine Fever Outbreaks: Implications for Post-Outbreak Control and Eradication

**DOI:** 10.3390/vetsci13040394

**Published:** 2026-04-17

**Authors:** Chi Ma, Wenfei Zhang

**Affiliations:** 1 School of Management, Hubei University of Chinese Medicine, Wuhan 430070, China; machi@webmail.hzau.edu.cn; 2 College of Humanities & Social Sciences, Huazhong Agricultural University, Wuhan 430070, China

**Keywords:** African swine fever, farmer behavior, consumer behavior, disease control, disease eradication, risk communication, spatial spillover

## Abstract

African swine fever (ASF) remains a major challenge for global animal disease prevention and control. Public sentiment during ASF, along with farmer and consumer behavior, are underexplored factors in ASF control. This study examines how public sentiment influences farmers’ risk perceptions and consumers’ behavioral responses, including consumption substitution intention and pork price expectations, and assesses the implications of these behaviors for disease control effectiveness. Using provincial panel data from China (June 2021–November 2022), sentiment analysis of 1.19 million Weibo posts, and a micro-level survey of 920 farmers, we combine panel regression, spatial econometric analysis, and micro-level behavioral evidence. Results show that public sentiment significantly elevates farmers’ risk perception, which may influence reporting decisions, marketing timing, and biosecurity investment, thereby increasing the complexity of surveillance and disease control. Sentiment intensifies substitution intentions and shapes pork price expectations, leading to reduced demand for formally marketed pork and potential shifts to lower-traceability or less-inspected channels. Spatial analysis indicates that the half-decay distance for amplifying ASF risk via sentiment is about 1300 km, providing parameters for cross-jurisdictional coordination. These findings support integrating socio-behavioral indicators into veterinary early warning systems and designing targeted disease risk communication under a broader One Health framework.

## Simple Summary

African swine fever (ASF) is a devastating disease affecting pigs worldwide, threatening livestock systems. While traditional control efforts focus on the virus itself, the influence of public sentiment and behavior on disease spread remains poorly understood. This study examines how public sentiment during ASF outbreaks affects farmers’ risk perceptions and consumers’ purchasing decisions, and how these behavioral changes impact disease control efforts. By combining social media sentiment analysis, provincial-level data, and a micro-level survey of 920 farmers, we find that negative public sentiment increases farmers’ risk perception, potentially leading to behaviors that affect the timeliness of disease reporting and the continuity of biosecurity practices, such as delayed reporting or earlier marketing decisions under uncertainty. Consumer perception and behavior also drive shifts away from formal pork markets toward channels with lower traceability or inspection visibility, creating challenges for veterinary inspection and timely risk identification. These effects show spatial spillovers, with a half-decay distance of about 1300 km, highlighting the need for coordinated cross-jurisdictional responses. Our findings suggest that integrating behavioral indicators into veterinary early warning systems and strengthening targeted disease risk communication for farmers and consumers should be integral components of ASF prevention and control under a broader One Health approach.

## 1. Introduction

The continued global spread of African swine fever (ASF), a devastating transboundary animal disease, poses a profound and persistent threat to animal health, biosecurity, and the stability of global livestock systems. The State of the World’s Animal Health 2025 released by the World Organisation for Animal Health indicates that from January 2024 to May 2025, 51 countries and territories have reported a total of 14,918 ASF outbreaks (3678 in domestic pigs and 11,240 in wildlife), resulting in 386,089 reported cases, with 605,225 animal losses in domestic pigs [1]. As the world’s largest producer, importer, and consumer of pork, China has been heavily affected by ASF since its first reported outbreak in 2018. The high mortality rate, contagiousness, and lack of a vaccine for ASF have severely affected China’s swine industry, leading to a cliff-like decline in China’s pig production capacity, with approximately 150 to 225 million pigs dying or being culled due to disease, accounting for 25% of global pig production [2]. ASF has led farmers to stop pig production or reduce their production scale, resulting in decreased pig stocking and replenishment, hindering the recovery of pig production and imposing severe livelihood losses on farmers [3]. Meanwhile, public confidence in pork consumption has declined, triggering collective panic among consumers, and the proportion of pork in the diet has continued to decrease. This drastic supply shock, compounded by shifts in consumer confidence, has led to significant adjustments across the pig industry chain [4,5,6]. Before effective vaccines are widely available, strict biosecurity measures (such as disinfection and transportation control) remain the cornerstone of ASF prevention and control [7]. Although ASF is not zoonotic, its impacts extend beyond animal health to farmers’ livelihoods, consumer confidence, mental well-being, and market stability [8]. This underscores the urgent need for a broader One Health-based disease prevention and control mechanism that integrates animal health, socio-economic resilience, and coordinated veterinary governance [9].

The digital age has transformed risk communication during animal disease crises. After the outbreak of ASF, social media platforms such as Weibo, Douyin, and Toutiao have become the main channels for information dissemination and emotional contagion during ASF events, profoundly influencing public risk perception [10]. These platforms not only accelerate the circulation of outbreak-related information but also amplify emotional reactions, shaping how farmers and consumers interpret ASF risk. In response to this changing information landscape, China’s Ministry of Agriculture and Rural Affairs (MARA) has implemented a series of public awareness campaigns, including regular epidemic bulletins, information initiatives, and reporting hotlines, to educate farmers and the public about ASF risks and proper biosecurity practices [11,12]. However, the reach and effectiveness of these official communications increasingly depend on how they interact with fast-moving online discussions, informal narratives, and emotionally charged content on digital platforms [11,13]. The Social Amplification of Risk Framework (SARF) offers one way to conceptualize this process: it suggests that when ASF risk signals encounter societal and individual “amplification stations,” public concern intensifies and behavioral responses follow [14]. Social media serves as a key amplification station in this chain. Individuals receive risk signals about ASF through social media and share their emotional states regarding ASF events. The psychological mechanisms of ASF at the individual level are influenced and guided by collective emotions, while social mechanisms at the overall level reshape public sentiment toward ASF through individual psychological judgments and retransmission [15]. Emotional cues embedded in interactive online content about ASF substantially boost the reach and impact of such information. The affective states and behavioral stances displayed by others on social media, whether consciously or unconsciously, can shape an individual’s emotional responses and actions [16]. In particular, “highly aroused” emotions and negative emotions can promote information-sharing behaviors, thereby facilitating the mutual transmission and wide dissemination of emotions in the online environment [17]. Importantly, in China, platforms such as Weibo, Douyin, and WeChat play distinct roles: Weibo serves as a public forum for rapid information diffusion, Douyin reaches wide audiences through short-video content, and WeChat functions as a closed, trust-based network among farmers. Together, they create a complex information ecosystem where official announcements, informal rumors, and emotional expressions interact to shape public sentiment. Within a short timeframe, these dynamics can generate concentrated public attention, intensifying societal scrutiny of animal disease governance. Such amplification can influence the behavioral responses of key stakeholders, farmers, and consumers alike in ways that may affect the effectiveness of official disease surveillance and biosecurity measures [18]. The resulting chain of social and behavioral impacts triggered by this amplification process may pose additional governance and communication challenges for veterinary authorities and MARA in designing ASF risk communication strategies, as well as for government departments in implementing ASF control and eradication efforts. In some contexts, these effects may be comparable in importance to the pathogen’s direct pathological effects [19,20].

The outbreak of ASF results in massive swine mortality, and the virus’s high transmissibility inflicts severe losses on the swine industry. The diffusion of negative emotions among farmers triggers panic. When farmers perceive anticipated farming risks as high, their risk perception of the epidemic intensifies, leading them to adopt a “wait-and-see” approach [21]. They only make decisions to resume production when the anticipated farming risks are deemed manageable [22]. Concurrently, under the constraints imposed by ASF, the diffusion of negative public sentiment amplifies uncertainty in the pork market, increasing the likelihood that farmers form negative expectations about market risks [23]. When farmers anticipate a depressed market with continuously falling prices, their willingness to resume production may diminish significantly, leading them to consider scaling back production or even exiting the swine industry altogether [24]. The amplifying effect of negative public sentiment on farmers’ risk perception directly reduces their willingness and subsequent actions to resume production following an epidemic. A more profound risk is that heightened risk perception among farmers may lead some producers to adopt short-term adjustment strategies, including delayed reporting, earlier marketing of pigs under uncertainty, or temporary reductions in on-farm biosecurity spending, which may affect the timeliness and completeness of disease surveillance [25,26]. These responses may reduce the sensitivity of veterinary surveillance and place additional pressure on existing biosecurity systems, potentially extending the window for undetected viral circulation. At this stage, field veterinarians play a pivotal role as the frontline link between the government and farmers. Through outbreak detection, reporting guidance, on-farm biosecurity support, and communication of support policies [27], they help farmers understand official requirements and facilitate alignment between policy implementation and on-farm practice [28].

Evidence from behavioral economics underscores the importance of public sentiment in shaping choices made under uncertainty and risk [29]. When transmitted rapidly through social media, public sentiment tends to amplify the risks associated with ASF. This amplification operates through information dissemination and emotional contagion: negative sentiment circulating on platforms such as Weibo and Douyin heightens consumers’ awareness of ASF risks and pork safety concerns, exerting a subtle yet meaningful effect on their intentions to substitute pork for other meats and on their expectations for pork prices. These shifts in turn modify consumer behavior and contribute to excessive fluctuations in pork market demand [30]. When the perceived risk of purchasing pork outweighs its perceived value, consumers often avoid pork altogether to reduce perceived market and product safety concerns [31]. This avoidance frequently leads them to select other protein sources, such as beef, mutton, or poultry, thereby forming substitution intentions for protein consumption [32,33,34]. The Theory of Planned Behavior explains how these intentions subsequently guide actual pork purchasing behavior [35]. From an animal disease prevention and control perspective, this shift in consumption behavior may engender two potential risks: first, the oversupply and stockpiling of pork products in formal market channels, which, if improperly handled, may create biosecurity management challenges; second, some consumers may prefer channels with lower transaction costs, where inspection visibility and traceability may be limited, potentially affecting the coverage of formal veterinary oversight [26,36]. Negative affective signals embedded in public sentiment may also foster pessimistic expectations about pork prices among consumers. The Theory of Bounded Rationality Expectations describes these forecasts as occupying a space between fully rational and completely irrational expectations. Rather than processing all available information, boundedly rational consumers rely partly on psychological factors, such as instinct and intuition, when forming expectations [37]. These expectations then meaningfully shape their decisions about pork consumption [38]. In this context, MARA plays an important role in supporting public understanding and market stability by delivering timely, authoritative, and science-based communication on the same digital platforms where consumers form their perceptions [39]. By providing official updates, pork safety guidance, and outbreak-related clarifications through channels such as Weibo and Douyin, MARA can help reduce rumor-driven panic and support continued use of formal market channels subject to veterinary inspection.

The cross-regional transmission pattern of ASF constitutes a fundamental challenge for veterinary disease control, as the virus readily crosses administrative boundaries through wild boar movement, livestock trade, and contaminated products. Crucially, in the digital age, the biological spatial transmission of ASF is paralleled by the cross-regional spread of public sentiment via social media platforms, resulting in a significant amplification of animal disease risks across geographical boundaries [40]. Public sentiment, through its signal-transmission function and emotional contagion, amplifies cross-regional risk for ASF [41], influencing the perceptions and behaviors of pork farmers and consumers far beyond the outbreak epicenter. This socially mediated spatial propagation of risk can, in turn, generate spatial spillover effects on pork market risk, with cascading implications for the resilience and stability of livestock systems. Understanding the spatial decay boundary of these spillover effects, namely the distance threshold beyond which such risk amplification dissipates, is therefore critical for designing spatially targeted veterinary surveillance, contingency planning, and cross-jurisdictional coordination mechanisms under a broader One Health framework that accounts for socio-behavioral and socio-economic dimensions of ASF risk [42].

In summary, the effective control of ASF extends beyond traditional veterinary measures to encompass the complex interplay between biological transmission and human behavioral responses in the digital information environment. Public sentiment, amplified through social media, serves as a critical force that can either reinforce or undermine official disease control efforts by shaping the risk perceptions and coping behaviors of farmers and consumers alike. These behavioral responses, ranging from changes in reporting and marketing decisions among farmers to consumption substitution and price expectations among consumers, carry direct implications for veterinary surveillance, biosecurity integrity, and risk management effectiveness. As such, this study investigates the social amplification of ASF risk and its effects on stakeholder behavior and the pork market, combining provincial panel data with a micro-level survey used as supplementary behavioral evidence. This design allows us to examine both the association between public sentiment and key behavioral outcomes at the macro level and the practical mechanisms through which these dynamics may affect veterinary governance, thereby providing empirical support for integrating socio-behavioral surveillance into veterinary early warning systems and for designing spatially coordinated disease control strategies under a broader One Health framework.

## 2. Materials and Methods

### 2.1. Data Sources

The dataset employed in this study comprises 30 Chinese provinces (including municipalities and autonomous regions) over the period June 2021–November 2022, excluding Tibet. ASF data are retrieved from the Official Veterinary Bulletin published by China’s Ministry of Agriculture and Rural Affairs (http://www.moa.gov.cn/gk/sygb/) (accessed on 3 April 2026). Indicators for public sentiment, consumption substitution intention, pork price expectations, and farmers’ risk perception are derived from social media posts gathered through programmed web crawlers and subsequently processed using natural language processing techniques for text classification and sentiment analysis. Price data are obtained from Bric Agricultural Big Data (http://www.chinabric.com/) (accessed on 3 April 2026). In addition, we draw on a survey of 920 farmers conducted in ASF-affected regions across Henan, Hubei, and Hunan provinces. The survey was conducted using stratified random sampling across 108 sample villages. The questionnaire collected information on household demographics, pig production practices, ASF exposure at both village and household levels, sources of information, perceived influence of public sentiment on production decisions, attitudes toward government compensation policies, and future farming intentions. These survey data are used to interpret mechanisms and provide supplementary evidence for the province-level findings, particularly regarding the roles of government departments, field veterinarians, social media platforms, and MARA in shaping farmer behavior.

### 2.2. Variable Selection

#### 2.2.1. Public Sentiment

With its massive user base, Sina Weibo ranks among the top social media platforms in China. It plays a key role in allowing the public to voice and exchange emotions during ASF, and it also serves as a “central source for information on unexpected ASF events.” Using Python 3.7.0 (Python Software Foundation, Beaverton, OR, USA) and Selenium 3.6.0 (Software Freedom Conservancy, Brooklyn, NY, USA), we developed a web crawler to collect individual users’ Weibo posts containing the keyword “African swine fever” from 1 June 2021 to 17 November 2022. After filtering out posts consisting solely of emoticons, links, duplicates, advertisements, or irrelevant content, the final corpus contained 1,198,438 posts.

Following data cleaning, the Weibo texts are transformed into document embeddings using the doc2vec model, and a CNN-based neural network is then applied to classify public sentiment. Based on Ekman’s classic framework for classifying emotions, this research identifies six types of emotions expressed by individuals on Weibo: anger, fear, surprise, sadness, neutrality, and joy [43]. Additionally, the two-dimensional valence–arousal model is used to assess public sentiment. Considering the diverse impact that emotions have on the spread of content on social media, public sentiment is categorized as follows: positive high-arousal emotions, which indicate pleasant and energetic responses to ASF events like joy, are assigned a value of 2; negative high-arousal emotions, denoting intense negative reactions such as anger, fear and surprise regarding ASF, are assigned a value of −2; negative low-arousal emotions reflect feelings of dissatisfaction and despair in the face of ASF challenges, such as sadness, and are allocated a value of −1; and a neutral category is used for blog posts that do not clearly exhibit any particular emotional feature, and is assigned a value of 0.

Finally, following the approach of Lu and Chen [44], we construct a monthly relative negative public sentiment index for each province using the formula:

(1) $$Pubsent_{it}=\frac{1+N_{it}^{NEG}}{1+N_{it}^{POS}}$$

where $N_{it}^{NEG}$ denotes the number of negative Weibo posts concerning ASF in province *i* during month *t*, and $N_{it}^{POS}$ denotes the corresponding number of positive posts. The resulting index ($Pubsent_{it}$) serves as our proxy for public sentiment.

#### 2.2.2. Farmers’ Risk Perception

Farmers’ risk perception refers to the subjective attitude and judgment of farmers regarding the risks of ASF. To construct this indicator, we collect comment texts from individual Weibo users discussing ASF between June 2021 and November 2022. From these comments, we extract content specifically related to farmers’ perceptions of disease risk. Only comments that refer to farmers’ ASF-related production and disease risks are retained for index construction. Each comment undergoes both text analysis and sentiment analysis, after which we apply Equation (1) to derive a province-level monthly measure of farmers’ risk perception.

#### 2.2.3. Consumption Substitution Intention

Consumption substitution intention reflects consumers’ propensity to replace pork with other meats. We extract from Weibo comments any discussion of reducing pork intake in favor of alternative protein sources. Only comments expressing an intention to reduce pork consumption or substitute pork with other meats are included in this indicator. Each comment is processed through text and sentiment analysis, and the index of consumption substitution intention is measured by Equation (1). This indicator captures changes in consumers’ stated intentions rather than realized purchases.

#### 2.2.4. Pork Price Expectations

Pork price expectations represent consumers’ subjective forecasts of future pork prices. We extract Weibo comments that mention expectations about pork prices and analyze them using text and sentiment techniques. Only comments containing explicit expectations regarding future pork price movements are retained. Equation (1) provides the monthly provincial indicator for pork price expectations. Together with consumption substitution intention, this variable captures the market-relevant behavioral response of consumers to public sentiment related to ASF.

#### 2.2.5. Pork Market Risks

We use the market price of deboned, skinless pork as the basis for measuring pork market risk. To remove the influence of inflation and seasonality, all price series are first deflated by the Consumer Price Index (with June 2021 as the base period) and then seasonally adjusted using the X-12-ARIMA seasonal adjustment program (U.S. Census Bureau, Washington, DC, USA). We then take natural logarithms of the adjusted data, compute first-order differences, and multiply the result by 100. Finally, we take the absolute value of this transformed series to obtain the pork market risk measure:

(2) $$Pork_{it}=\left|100\times \ln(p_{t}/p_{t-1})\right|$$

where $p_{t}$ represents the pork price in period *t*.

#### 2.2.6. Control Variables

For all continuous control variables, we apply the same volatility transformation, i.e., |100 × ∆ln(variable)|. Price-related controls are also deflated using the CPI (June 2021 base) and seasonally adjusted with the X-12 procedure. The variables are as follows: (1) Pig price volatility (Pig), defined as price fluctuations of live slaughter pigs, representing a midstream segment of the industry chain. (2) Piglet price volatility (Piglet), defined as price fluctuations of piglets, capturing upstream conditions. (3) Corn price volatility (Corn), defined as volatility in corn prices, given that corn constitutes over 50% of pig feed. (4) Soybean meal price volatility (Sbm), defined as fluctuations in soybean meal prices, another key feed input. (5) Beef price volatility (Beef), defined as the volatility of boneless beef prices, serving as a substitute protein. (6) Mutton price volatility (Mutton), defined as the volatility of bone-in mutton prices. (7) Broiler price volatility (Broiler), defined as the volatility of dressed chicken prices. (8) Disposable income of urban residents (Income), defined as volatility in urban disposable income, reflecting consumer purchasing power. (9) The number of live pigs sold (Supply), measured as the logarithm of monthly live pig sales, is used to capture regional supply conditions. These controls are included to account for upstream production costs, downstream substitution effects, and market conditions that may jointly influence stakeholder behavior and pork market risk. Descriptive statistics for all variables appear in Table 1.

### 2.3. Methods

#### 2.3.1. Benchmark Model

To examine how public sentiment shapes stakeholders’ risk perceptions and behavioral responses during ASF, we specify the following baseline regression model:

(3) $$Y_{it}=\varphi_{0}+\varphi_{1}Pubsent_{it}+\varphi_{2}Controls_{it}+\varepsilon_{it}$$

where *i* and *t* index province and month, respectively; the dependent variable $Y_{it}$ indicates farmers’ risk perception, consumption substitution intention, and pork price expectations; $Pubsent_{it}$ denotes our measure of public sentiment regarding ASF; and $Controls_{it}$ represents covariates influencing stakeholders’ risk perceptions and behavioral responses. All benchmark regressions include province fixed effects and month fixed effects to control for time-invariant provincial heterogeneity and common temporal shocks.

#### 2.3.2. Spatial Panel Durbin Model

Spatial units interact with neighboring units through various connections, leading to spatial dependence and potential spillover effects. Public sentiment is likely to exhibit spatial externalities, and pork market risks may be correlated across neighboring provinces. Consequently, when analyzing the impact of public sentiment on pork market risks during ASF, it is important to incorporate spatial interactions. The Spatial Panel Durbin Model (SPDM) addresses this by including spatial lags of both the dependent variable and the explanatory variables. We employ the SPDM because it can simultaneously capture local effects and cross-regional spillover effects of public sentiment and market variables, which is essential for identifying the spatial range of sentiment-driven ASF risk amplification. We specify the following SPDM:

(4) $$Pork_{it}=\alpha +\rho \sum_{j=1}^{N}\omega_{ij}Pork_{it}+\beta_{1}Pubsent_{it}+\beta_{2}X_{it}+\theta_{1}\sum_{j=1}^{N}\omega_{ij}Pubsent_{it}+\theta_{2}\sum_{j=1}^{N}\omega_{ij}X_{it}+\mu_{i}+\psi_{t}+\varepsilon_{it}$$

where ρ captures the spatial autoregressive coefficient, indicating how local pork market risks affect those in neighboring regions; Pubsent is the public sentiment matrix; *X* represents the matrix of control variables; θ is the regression coefficient of the spatial lag term; *w* is the spatial geographical weight matrix; α is the model intercept term; $\mu_{i}$ and $\psi_{t}$ denote individual and time fixed effects; and $\varepsilon_{it}$ is the random error term.

#### 2.3.3. Spatial Weight Matrix

Spatial dependence and spillover effects often stem from geographical proximity. To capture this, we construct a spatial weight matrix based on the inverse Euclidean distance between provincial centroids, which are derived from longitude and latitude coordinates. The matrix elements are defined as:

(5) $$W_{ij}(1)=\left\{\begin{array}{ll} 1/d_{ij} & d_{ij}<d\\ 0 & d_{ij}\geq d \end{array}\right.$$

where $d_{ij}$ represents the distance between provinces i and j.

Because regional economic conditions influence consumption levels and thereby affect pork demand, we also employ a gravity-based weight matrix that incorporates both geographic distance and economic scale (proxied by per capita GDP). This matrix takes the form:

(6) $$W_{ij}(2)=\left\{\begin{array}{ll} (\bar{E_{I}}\times \bar{E_{j}})/d_{ij}^{2} & i\neq j\\ 0 & i=j \end{array}\right.$$

where $\bar{E_{i}}$ and $\bar{E_{j}}$ denote the per capita GDP in provinces *i* and *j*. This specification assumes that spatial linkages depend not only on physical proximity but also on the relative economic size of the regions involved.

## 3. Results

### 3.1. Public Sentiment During ASF and Farmers’ Risk Perception

Columns (1) to (3) of Table 2 report the estimation results for Equation (3). The emotional diffusion of negative public sentiment during ASF outbreaks significantly influences farmers’ risk perception, a critical determinant of on-farm biosecurity compliance and disease reporting behavior. Column (1) shows that the impact coefficient of emotional diffusion on farmers’ risk perception is significantly positive at the 5% level, indicating that negative emotional diffusion during ASF outbreaks increases farmers’ risk perception. The emotional diffusion of ASF continuously feeds back into and circulates through social media, shaping new risk perceptions among farmers. From a veterinary disease control perspective, heightened risk perception among farmers carries dual implications: on one hand, it may motivate enhanced biosecurity vigilance; on the other hand, when risk perception exceeds a certain threshold, some farmers may prioritize short-term production continuity, leading to delayed reporting, earlier marketing decisions, or temporary adjustments in biosecurity investment, which can affect the completeness of surveillance data [26]. These responses may reduce the timeliness of veterinary supervision and increase operational challenges in maintaining consistent biosecurity standards. This dynamic highlights the importance of clear support arrangements and effective frontline communication, with MARA coordinating policy design and field veterinarians supporting farmers’ understanding of reporting requirements and movement management measures [28].

### 3.2. Public Sentiment During ASF and Consumption Substitution Intention

This section examines the impact of public sentiment on consumers’ consumption substitution intention during ASF outbreaks. Consumption substitution intention is entered as the dependent variable in the regression, with results presented in Table 2. As column (2) indicates, public sentiment positively affects consumption substitution intention, meaning that exposed consumers tend to reduce pork purchases and turn to alternative protein sources. The underlying mechanism is that public sentiment conveys emotional signals about ASF to consumers. Through emotional contagion, individuals perceive the risks associated with ASF as more severe, prompting them to weigh risk aversion against risk preference. When the perceived safety risks of pork consumption exceed its expected benefits, consumers are more likely to choose other meats, thereby increasing their substitution intention. To support informed decision-making, MARA can continue to use digital platforms to disseminate factual, science-based content, including clear information that ASF is not zoonotic and that pork from regulated slaughter and inspection systems remains safe for consumption [13,36]. Changes in consumption behavior have a direct impact on veterinary risk management. First, reduced demand for formally marketed pork may lead to product stockpiling; if improperly stored or handled in downstream circulation, these products could increase biosecurity management pressure within the supply chain. Second, when consumers choose channels with lower inspection visibility and weaker traceability, this may reduce the coverage of formal veterinary oversight and increase the difficulty of timely risk identification. Therefore, the veterinary department can strengthen biosecurity oversight in cold-chain and distribution links, while market regulators and MARA can further standardize slaughtering and trading practices and improve the accessibility and credibility of formal market channels for consumers.

### 3.3. Public Sentiment During ASF and Pork Price Expectations

The estimated effect of public sentiment on consumers’ pork price expectations in the context of ASF outbreaks is shown in column (3) of Table 2. The coefficient for public sentiment is 0.0671 and statistically significant at the 1% level, confirming that public sentiment amplifies pork price expectations. More specifically, emotional contagion leads boundedly rational consumers to form pessimistic expectations about the direction of pork prices. When consumers expect sustained price increases due to supply shortages, their purchasing patterns may shift, potentially leading to panic buying or stockpiling of pork products. More critically, for farmers, pessimistic price expectations combined with heightened risk perception may encourage short-term sales decisions before full health verification is completed, thereby increasing the difficulty of preventing undetected viral circulation and cross-farm transmission. In this context, veterinary authorities should provide transparent, data-driven updates on outbreak status, pork supply, and price trends to counter misinformation and calm irrational expectations. Meanwhile, MARA can leverage its official channels, such as press releases and social media accounts, to communicate price-stabilization measures, for example, by releasing frozen pork reserves. Such coordinated risk communication helps anchor market expectations, support orderly market adjustment, and preserve the integrity of formal marketing channels, ultimately supporting post-outbreak control and eradication efforts.

### 3.4. Empirical Evidence of Farmer Behavioral Responses to ASF

To complement the macro-level public sentiment analysis, we draw on a survey of 920 farmers conducted in ASF-affected regions across Henan, Hubei, and Hunan provinces. The survey results are presented in Table 3, and the detailed data sources and sampling methods are described in Section 2.1. This micro-level survey provides supplementary evidence on the behavioral pathways hypothesized in our macro-level sentiment analysis, documenting key farmer responses, including reporting delays, accelerated sales, production exit, and susceptibility to social media influence, that may affect ASF surveillance, biosecurity, and post-outbreak eradication efforts. Crucially, the survey also helps clarify the practical relevance of government departments, field veterinarians, social media, and MARA in shaping farmers’ responses during ASF outbreaks.

Epidemic exposure and underreporting: The survey reveals a substantial gap between community-level and household-level exposure to outbreaks. While 45.3% of villages experienced ASF outbreaks, only 15.9% of households reported that their own farms were affected—a discrepancy of 29.4 percentage points. This gap suggests that delays or omissions in formal reporting may occur under certain circumstances, potentially affecting the completeness of ASF surveillance data and reducing the timeliness of response measures [26]. When infections are not reported in a timely manner, outbreaks may remain in the production system longer than expected, delaying response measures and increasing the risk of wider spread. The magnitude of this gap indicates that underreporting may represent an important vulnerability in ASF control. Among affected households, average herd size dropped from 4.0 head pre-outbreak to 0.165 head post-outbreak, a 24-fold reduction, demonstrating the devastating impact of ASF on farmers’ production capacity. The near-total collapse of pig inventories among affected households means that disease reporting may involve considerable short-term economic pressure for farmers. This highlights the importance of making compensation arrangements more predictable, timely, and clearly communicated. This finding indicates that farmers’ understanding of compensation policy, together with the effectiveness of field-level communication and implementation, may influence their willingness to report in a timely manner.

Threshold effects in pig offtake patterns: The temporal pattern of pig offtake—the number of pigs sold or removed from farms—exhibits a clear threshold effect with direct implications for ASF control. In the year preceding the ASF outbreak, average annual pig offtake per household was 11.6 head. During the outbreak year, offtake increased to 13.4 head (+15.8%). This increase reflects two co-occurring behavioral drivers. First, farmers facing uncertainty and the fear of infection may accelerate sales to reduce potential losses under uncertain market and epidemic conditions. According to Prospect Theory, anticipated losses may shape decision-making under uncertainty, increasing the likelihood that farmers prioritize immediate stability in production arrangements. Second, farmers anticipating higher pork prices due to supply shortages may accelerate sales to capitalize on expected profits. From a veterinary perspective, rapid sales under market pressure may increase the likelihood that pigs enter circulation before health conditions are fully assessed or relevant procedures are completed, thereby increasing the difficulty of preventing undetected viral circulation [26]. In the year following the outbreak, offtake collapsed to 6.6 head, a 50.9% reduction relative to the outbreak year. This sharp contraction reflects depletion of available stock following accelerated sales during the outbreak period, compounded by widespread exit from production, with 67.9% of farmers reported no intention to resume pig farming. These patterns reveal an important coordination issue for veterinary authorities: when farmers perceive rapid offloading as their only economic survival strategy, enforcing movement restrictions becomes difficult. Here, clearer compensation arrangements and timely, science-based communication may help farmers better understand official response procedures and make production decisions that are more consistent with disease control objectives. Field veterinarians can then translate these policies into practical, direct farm-level guidance.

Information channels and sentiment influence: The survey reveals the relative importance of different information channels in shaping farmer behavior during the ASF outbreak. When farmers ranked the importance of various information sources (1 = most important, 6 = least important), social media and word of mouth ranked as the two most important channels, surpassing traditional media. Farmers reported moderate attention to ASF-related public sentiment on social media (mean 3.09 out of 5), and 43.7% reported that public sentiment significantly influenced their production decisions (31.9% “comparatively influential”, 11.8% “very influential”). Nearly half of all farmers reported that public sentiment directly shapes their production decisions. This finding points to a critical role for social media in ASF information dissemination: digital platforms have become the primary channels through which farmers receive information and form perceptions, and emotionally charged content can spread rapidly, magnifying the risks of ASF and driving fear behaviors. For MARA, this suggests that effective risk communication cannot rely solely on traditional channels such as television and newspapers. Actively leveraging digital platforms to shape the information environment can effectively curb the escalation of ASF risks and support farmers in making more adaptive responses.

Risk perception and behavioral intentions: Farmers’ plans confirm the behavioral patterns documented above. Only 32.1% of farmers reported plans to raise pigs in the 1–2 years following the ASF outbreak, indicating widespread industry exit. Among farmers planning to reduce herd size, high ASF risk consistently ranks as the most important factor driving their decision. Similarly, among those not planning to resume pig farming, high ASF risk emerges as the second most influential reason. These findings demonstrate that perceived ASF risk is a primary determinant of farmers’ decisions to exit or scale back production. When farmers exit production, they may seek to dispose of remaining stock through channels with lower transaction costs, where inspection visibility and traceability may be relatively limited. This can increase the difficulty of tracking susceptible pigs and maintaining complete surveillance coverage. Moreover, the role of ASF-related risk perception in shaping behavioral intentions underscores the importance of maintaining farmer trust in veterinary authorities. Overall, the survey evidence suggests that ASF-related risk perception is closely associated with farmers’ decisions regarding herd adjustment and continued production. During this process, transparent communication and effective epidemic response by government departments and field veterinarians are important for maintaining farmer confidence and supporting the stability of the producer base.

### 3.5. Spatial Spillover Effects of Public Sentiment During ASF on Pork Market Risks

To examine whether the influence of public sentiment during ASF extends across regional boundaries, we estimate the SPDM with fixed effects. The results are presented in Table 4. Column (1) presents the inverse-distance-matrix estimation results. The direct effect coefficient for public sentiment is 0.3441 (*p* < 0.01), indicating that local sentiment significantly increases local pork market risk. The indirect effect is estimated at 0.6222 and is also significant at the 1% level, implying that sentiment spillovers elevate pork market risks in adjacent regions. This spatial spillover can be attributed to two interconnected processes. First, the virus itself exhibits inherent spatial diffusion through wild boar movement, livestock trade, and contaminated product flows—creating a biological risk gradient that decays with distance. Second, and critically, public sentiment transmits emotional signals of ASF risk via social media across administrative boundaries, influencing the perceptions and behaviors of pork consumers and farmers in neighboring regions before the virus physically arrives. This dual-process spatial dynamic–biological transmission, paralleled by socially mediated risk amplification, creates a compounded threat to livestock systems. These results indicate that the effects of public sentiment are not confined to the province in which the outbreak-related information originates. This relates to cross-regional coordination between government departments and MARA, such as whether field veterinarians in neighboring areas coordinate their risk communication strategies and whether MARA breaks down administrative boundaries to facilitate information sharing across provinces.

Column (2) of Table 4 reports the spatial spillover estimates obtained with the gravitational matrix, recognizing that regional economic conditions influence pork market supply and demand. Both the direct and indirect effects of public sentiment remain significantly positive, although the magnitude of the indirect effect is somewhat smaller than in column (1). The spatial dynamic panel data (SDPD) model provides a more rigorous approach to addressing endogeneity concerns arising from spatial weights than conventional static spatial panel models. Column (3) displays the SDPD model results based on the inverse distance matrix. The spatial lag term coefficient for pork market risk is positive and significant (*p* < 0.10), while the spatial spillover coefficient for public sentiment declines further to 0.5687. This attenuation suggests that static panel models tend to inflate the estimated spillover effects of public sentiment on pork market risk. Column (4) presents the SDPD model estimates using the gravitational matrix; both direct and indirect effects of public sentiment continue to be significantly positive. The consistent significance of spatial spillover effects across multiple model specifications underscores a key insight for veterinary disease control: risk communication and behavioral interventions need to be coordinated across jurisdictional boundaries, as the social influence of an outbreak often extends beyond the immediate epidemiological center.

### 3.6. Spatial Decay Boundary of the Spatial Spillover Effect

According to Tobler’s First Law of Geography, spatial correlation weakens as the distance between provinces increases. The transmission range of the ASF virus, the attenuation characteristics of information in spatial transmission, and regional cultural environment differences may lead to spatial influences on public sentiment, with certain geographical attenuation and decay boundaries. Identifying this decay boundary has direct implications for defining the spatial scope of cross-jurisdictional surveillance coordination and emergency response zoning. To examine how the spillover effect of public sentiment on pork market risk varies with distance, Equation (5) is used to construct a distance-threshold spatial matrix. In our sample, the smallest inter-provincial distance is 75.01 km, and the largest is 3597 km. Accordingly, we define the initial threshold distance for the spatial weight matrix as 100 km and increment it in steps of 100 km. Because the number of province pairs with spatial links retained in the weight matrix decreases sharply beyond 2800 km, and the resulting spillover estimates become unstable due to outlier influence (which introduces substantial noise), this analysis only includes results for distances up to 2800 km. Using the distance-threshold spatial weight matrix, we then reapply the SPDM to estimate the spillover effects of public sentiment on pork market risk for each distance interval.

Table 5 summarizes how the spatial spillover effects of public sentiment during ASF change with increasing geographical distance. As distance increases, spatial spillover effects exhibit clear distance-decay characteristics, falling into three distinct intervals. The first interval covers distances up to 1100 km, where spillover coefficients reach their peak values, generally spanning one to three adjacent provinces. This zone represents the core area of ASF risk amplification, where public sentiment directly influences the perceptions and behaviors of nearby pork farmers and consumers through signal transmission and emotional contagion effects. From a veterinary standpoint, this 1100 km radius defines the critical scope within which MARA and veterinary authorities should prioritize coordinated risk communication and the implementation of a unified biosecurity information dissemination plan, as behavioral responses in these adjacent areas can significantly impact the effectiveness of regional disease control efforts. The second interval, covering 1100 km to 1600 km, witnesses a sharp decline in spatial spillover effects. The spatial spillover coefficient drops from its peak of 0.5548 to 0.2902, yielding a half-decay distance of 1300 km. This suggests that beyond 1300 km, the direct behavioral influence of sentiment originating from an outbreak epicenter diminishes substantially. This finding supports a tiered approach to cross-regional coordination, with intensified surveillance and information sharing within a 1300 km radius, while areas beyond may require different, locally tailored intervention strategies. In the third interval, beyond 1600 km, the coefficients become statistically insignificant and display erratic fluctuations. Thus, the spatial decay boundary for public sentiment lies at approximately 1600 km. Past this threshold, public sentiment exerts no measurable spillover effect on pork market risks in more distant regions. However, it is important to note that this does not imply the absence of biological transmission risk over such distances, as the ASF virus can still be transported through long-distance livestock trade or contaminated products. Rather, it suggests that beyond this boundary, risk communication and behavioral interventions should be designed based on local outbreak contexts rather than assuming direct spillover of sentiment from distant epicenters.

## 4. Discussion

This study finds that during ASF, public sentiment acts as a critical “amplification station” within the Social Amplification of Risk Framework (SARF), significantly amplifying ASF-related risks by shaping the risk perceptions and behavioral responses of farmers and consumers, two stakeholder groups whose decisions directly influence disease transmission dynamics and the effectiveness of veterinary control measures. This amplification unfolds through several distinct behavioral pathways, including heightened risk perception among farmers, stronger consumption substitution intention among consumers, and shifts in pork price expectations, each of which carries direct implications for veterinary disease control. These pathways are further supported by our micro-level survey, which documents the tangible outcomes of behaviors driven by ASF risk and public sentiment, including reporting delays, accelerated sales, and production adjustments, all of which may affect ASF control and eradication efforts.

From a veterinary perspective, the heightened farmers’ risk perception is particularly significant. While appropriate risk awareness is necessary for biosecurity compliance, excessive risk perception may lead some farmers to prioritize short-term production continuity over longer-term biosecurity planning, resulting in delayed reporting, earlier marketing decisions, or temporary reductions in biosecurity investment [21,45]. These responses may reduce the sensitivity of veterinary surveillance, making it more difficult to detect and interrupt viral persistence and circulation in a timely manner. In this process, government departments, particularly field veterinarians, occupy a pivotal position because they constitute the most immediate institutional interface between formal disease control systems and farmers’ day-to-day production decisions [27]. The role of field veterinarians extends beyond diagnosis, reporting, and quarantine implementation; it also includes helping farmers understand official control measures and improving communication between policy implementation and on-farm practice. When veterinary services provide timely guidance on reporting, support policies, and disease management, farmers are more likely to align their decisions with disease control objectives [45]. Thus, farmers’ perceptions of government departments, especially as experienced through field veterinarians, form an important mechanism through which public sentiment is translated into concrete behavioral outcomes.

The micro-level survey provides supplementary evidence consistent with this dynamic. The gap between village-level and household-level outbreak reports suggests that delays or omissions in reporting may occur under certain conditions, potentially affecting surveillance completeness and response timing. Moreover, the survey reveals a threshold pattern with direct implications for disease control. During the outbreak year, pig offtake increased amid heightened uncertainty, reflecting decisions to accelerate sales under perceived production risk. When ASF risk leads to high uncertainty about the survival of the livestock herd, production continuity becomes the primary concern for farmers, which may affect some farmers’ compliance with standard procedures. The subsequent collapse in offtake, coupled with the finding that epidemic risk ranks as the dominant factor in farmers’ decision to reduce herd size, reveals the endpoint of this trajectory: exit from production. Together, these patterns are consistent with a behavioral sequence linking elevated ASF risk perception to reporting delays, accelerated sales, and production exit, which may increase the complexity of eradication efforts by affecting movement management, surveillance coverage, and producer continuity [21]. Such withdrawal weakens both the producer base for sustained control and routine links between farmers and veterinary services. Reversing this trend requires more than emergency containment. Government departments can help reduce the perceived costs of compliance by providing clearer, more predictable post-outbreak support. Field veterinarians can contribute by offering timely farm-level guidance, strengthening communication with farmers, and translating control requirements into feasible recovery measures. At the same time, MARA has an important role in further improving the transparency, consistency, and accessibility of compensation, restocking, and recovery policies, thereby strengthening farmers’ confidence that continued production can remain economically viable while supporting effective disease control [46].

The amplification of ASF risk through consumer channels presents important implications for veterinary public health. Our findings demonstrate that negative sentiment enhances consumers’ consumption substitution intention and shapes pork price expectations. These behavioral shifts require attention in veterinary risk management: reduced demand for formally marketed pork may lead to product accumulation in cold chains; if improperly stored or handled in downstream circulation, these reserves can increase management pressure in the supply chain [47]. More critically, when consumers turn to procurement channels with lower inspection visibility and weaker traceability, the coverage of formal veterinary inspection may be reduced, creating additional challenges for surveillance and risk management [48]. To date, however, relatively limited attention has been paid to the link between veterinary risk management and consumer cognition and behavior. In this context, social media functions as both a risk amplifier and a potential governance tool [49]. MARA can continue to strengthen public awareness by disseminating timely, authoritative, and understandable information on ASF through the social media platforms where consumers obtain and exchange information [13]. Such communication is especially important for clarifying the distinction between ASF risk and pork consumption safety, improving public understanding, and maintaining confidence in regulated slaughter, inspection, and marketing systems.

The spatial spillover effects identified in this study appear to arise from the interaction between the biological spread of ASF and the cross-regional transmission of risk perception and behavioral responses through social media, thereby generating a broader threat to the livestock system. The half-decay distance is approximately 1300 km, and the clear spatial attenuation boundary provides evidence-based parameters for defining cross-jurisdictional surveillance coordination and emergency response zones. Within 1300 km, coordinated and consistent disease risk communication efforts [50], as well as simultaneous biosecurity information promotion, should be prioritized, as behavioral responses in these adjacent regions will significantly impact disease control effectiveness within the region.

Our findings offer actionable insights for veterinary authorities under a broader One Health paradigm that incorporates socio-behavioral and socio-economic dimensions of animal disease risk: First, establish a “Socio-Behavioral Risk Monitoring Index” that integrates public sentiment, farmer risk perception, and consumer behavioral indicators into existing animal disease early warning systems. This enhanced monitoring tool can offer early signals of behavioral change, such as delayed reporting, accelerated sales, or shifts in purchasing channels, thereby enabling more proactive risk communication and earlier policy adjustment before disease control becomes more difficult [51]. Second, the identified spatial decay boundary (1300 km half-distance) provides an evidence-based parameter for designing spatially targeted veterinary interventions and cross-jurisdictional coordination mechanisms. This supports a tiered model of regional cooperation, with strengthened monitoring and coordinated information dissemination within the core area, and more locally adapted strategies beyond it. Third, develop targeted risk communication strategies that address the specific behavioral pathways identified in this study, guide rational consumption choices, correct irrational price expectations, and provide farmers with clearer economic support arrangements and practical guidance, thereby better aligning production decisions with disease control objectives. Such communication efforts should be deployed rapidly, before sentiment polarization escalates, to preserve the integrity of biosecurity measures and veterinary surveillance systems.

## 5. Conclusions

This study extends the application of the Social Amplification of Risk Framework to the domain of transboundary animal disease control, demonstrating that the socio-behavioral dimensions of ASF outbreaks are not secondary considerations but integral components of effective veterinary governance. By integrating macro-level social media analytics with micro-level survey data, we provide complementary empirical evidence that public sentiment is closely associated with the risk perceptions and behavioral responses of both farmers and consumers, with important implications for the effectiveness of veterinary control and post-outbreak eradication measures. The main findings are as follows:

First, negative public sentiment during ASF outbreaks significantly increases farmers’ risk perception, with dual implications for disease control. While appropriate risk awareness supports biosecurity adherence, elevated perceptions may prompt some farmers to adopt short-term production adjustments, such as delayed reporting, earlier marketing, or temporary reductions in biosecurity investments. These responses reflect adaptive decision-making under uncertainty and highlight the need for support mechanisms that align farmer incentives with long-term disease control goals. The micro-level survey provides supplementary evidence of this pattern, with a measurable gap between community and household-level outbreak reporting, and epidemic risk cited as a key factor in production decisions. These findings indicate that the effectiveness of ASF control depends not only on epidemiological risk, but also on how farmers understand and experience the support and communication provided by government departments, especially field veterinarians.

Second, consumer behavioral responses to public sentiment constitute a distinct pathway influencing veterinary control systems. Public sentiment intensifies intentions to substitute pork with alternative proteins and shapes pessimistic price expectations. These responses may affect demand for pork marketed through formal channels, increase product management requirements in storage and cold-chain systems, and lead some consumers to prefer purchasing channels with lower traceability or inspection visibility. As a result, additional challenges may arise for veterinary oversight, supply-chain traceability, and timely risk identification. These mechanisms highlight that media, especially social media platforms, are an important channel through which ASF-related information reaches the public and influences consumer behavior. They also underscore the importance of timely, credible communication from government departments and MARA on ASF risk and pork consumption.

Third, the influence of public sentiment on pork market risk exhibits clear spatial spillover, with a half-decay distance of approximately 1300 km. This spatial structure reflects the parallel operation of two transmission processes: the physical movement of the virus via cross-border pig transport and the diffusion of risk perceptions through digital networks that transcend administrative borders. These findings provide empirical guidance for identifying zones requiring differentiated surveillance intensity, communication strategies, and inter-jurisdictional coordination protocols essential to ASF eradication.

Drawing from the above conclusions, we propose the following policy recommendations for veterinary governance under a broader One Health framework: First, integrate socio-behavioral indicators into veterinary surveillance systems. Real-time monitoring of farmer risk perception, consumption substitution intention, pork price expectations, and changes in public sentiment could serve as an early-warning tool for identifying situations in which reporting, marketing, or purchasing decisions may become less aligned with disease control needs. Such a system would enable veterinary authorities to intervene before behavioral disruptions undermine biosecurity and facilitate undetected viral circulation. In particular, government departments should use such signals to identify areas or producer groups that may require more targeted communication and support, and respond with clearer compensation arrangements and more transparent control procedures. Field veterinarians should translate these warnings into prompt, practice-oriented guidance and direct communication with producers. Second, operationalize spatial decay parameters in the cross-jurisdictional disease control coordination framework. Within the 1300 km core zone, veterinary authorities should prioritize harmonized surveillance protocols, synchronized movement restrictions, and coordinated risk communication campaigns. In the 1300–1600 km transition zone, enhanced monitoring with locally adapted strategies is indicated, while beyond 1600 km, interventions should be guided primarily by local outbreak status rather than assuming direct sentiment spillover from distant epicenters. Third, develop differentiated risk communication strategies for different stakeholder groups involved in animal diseases. For farmers, communication should emphasize clear reporting pathways, understandable compensation arrangements, and practical biosecurity guidance so as to encourage timely reporting and more prudent marketing decisions. For consumers, authorities should deliver science-based messaging through both official and social media channels to improve public understanding of pork safety, respond to inaccurate information in a timely manner, and reinforce the value of formal market channels that maintain veterinary inspection. MARA can continue to play a central role in public communication by emphasizing that ASF is an important animal disease affecting pig production, while also clearly explaining that pork that has passed regulated slaughter and inspection procedures is safe for consumers.

While this study strives for methodological rigor and robust conclusions, the complexity of the subject matter introduces certain limitations that warrant acknowledgment and future exploration. First, although this study identifies associations between public sentiment during ASF and stakeholder perceptions and behaviors at the aggregate level and uses a micro-level survey as supplementary evidence on relevant behavioral patterns, the survey was designed as a cross-sectional rather than a longitudinal panel study tracking the same households over time. This design limits the ability to observe how individual farmers’ risk perceptions evolve dynamically and how specific ASF-related behavioral decisions, such as outbreak reporting, biosecurity investments, or the movement of potentially infected pigs, are made sequentially in response to changing epidemic conditions. Future research should implement longitudinal panel surveys or field experiments within veterinary epidemiology to capture these dynamic processes. Such work would provide critical evidence for designing targeted interventions that address the behavioral drivers of ASF transmission at different stages of outbreak response and post-outbreak eradication. Second, the analysis relies on social media data collected from Chinese platforms, with Weibo being the primary source. While China represents the world’s largest pork market and provides a robust context for examining ASF-related risk amplification, the cultural specificity of sentiment expression, platform ecology, and institutional trust may influence the generalizability of specific quantitative parameters to other settings. Future research should conduct comparative studies across diverse socio-cultural and regulatory environments to examine how variations in veterinary governance structures, biosecurity cultures, and digital media landscapes moderate the relationship between public sentiment and stakeholder behaviors. Such cross-contextual validation would strengthen the evidence base for integrating socio-behavioral surveillance into broader global One Health frameworks for transboundary animal disease management.

## Figures and Tables

**Table 1:** Statistical characteristics.

Variable Name	Code	Mean	SD	Min	Max
Public sentiment	Pubsent	1.966	0.977	0.2520	5.929
Farmers’ risk perception	Frisk	0.031	0.492	−1	1
Consumption substitution intention	Substint	0.145	0.460	−1	1
Pork price expectations	Priceexp	0.412	0.412	−1	1
Pork market risks	Pork	7.653	6.189	0.0014	33.97
Pig price volatility	Pig	9.392	8.048	0.0568	33.38
Piglet price volatility	Piglet	13.27	9.079	0.0246	47.95
Corn price volatility	Corn	1.987	1.902	0.0065	9.789
Soybean meal price volatility	Sbm	3.250	4.191	0.0022	28.53
Beef price volatility	Beef	0.802	0.707	0.0015	5.746
Mutton price volatility	Mutton	1.273	1.122	0.0029	10.02
Broiler price volatility	Broiler	1.413	1.594	0	15.42
Disposable income of urban residents	Income	0.346	0.258	0.0003	2.306
The number of live pigs sold	Supply	4.170	1.383	0.728	6.242

Note: Pubsent is the monthly province-level relative negative public sentiment index for ASF. Frisk, Substint, and Priceexp are constructed from ASF-related Weibo comments using text classification and sentiment analysis. Price-related variables are deflated by the CPI (June 2021 = 100) and seasonally adjusted using the X-12-ARIMA seasonal adjustment program (U.S. Census Bureau, Washington, DC, USA).

**Table 2:** Benchmark regression results for the effects of public sentiment on stakeholder risk perceptions and behavioral responses.

Variables	(1) Farmers’ Risk Perception	(2) Consumption Substitution Intention	(3) Pork Price Expectations
Pubsent	0.0444 **	0.0912 ***	0.0671 ***
	(0.0170)	(0.0222)	(0.0132)
Pig	0.0034	−0.0006	0.0051 ***
	(0.0023)	(0.0022)	(0.0016)
Piglet	−0.0027 *	−0.0007	0.0007
	(0.0016)	(0.0022)	(0.0020)
Corn	0.0017	−0.0149 *	−0.0125
	(0.0127)	(0.0080)	(0.0088)
Sbm	−0.0330 ***	−0.0016	−0.0133 **
	(0.0063)	(0.0055)	(0.0049)
Beef	−0.0364	−0.0729	−0.0469
	(0.0397)	(0.0459)	(0.0291)
Mutton	−0.0827 ***	−0.0498 *	−0.0453 *
	(0.0258)	(0.0280)	(0.0246)
Broiler	−0.0135	0.0287 **	0.0133
	(0.0109)	(0.0107)	(0.0098)
Income	−0.2252 **	−0.0584	0.0179
	(0.0956)	(0.0829)	(0.0675)
Supply	−0.0001	0.0000	−0.0000
	(0.0001)	(0.0002)	(0.0001)
Cons	0.4735 ***	−0.0191	0.3144 ***
	(0.0406)	(0.0481)	(0.0378)
N	540	540	540
R^2^	0.1238	0.0811	0.0937

Note: Standard errors are shown in parentheses. ***, **, and * indicate statistically significant differences at the 1%, 5%, and 10% levels, respectively.

**Table 3:** Empirical evidence of farmer behavioral responses to African swine fever (ASF).

Indicator	N	Mean	SD	Min	Max
**Epidemic Exposure and Underreporting**					
Villages that experienced ASF outbreaks (%)	920	0.453	0.498	0	1
Households that experienced ASF outbreaks (%)	920	0.159	0.366	0	1
Pre-ASF herd size (affected households, head)	920	3.999	38.519	0	1000
Post-ASF herd size (affected households, head)	920	0.165	2.405	0	70
**Threshold Effects in Pig Offtake Patterns**					
One year pre-ASF (pig offtake per household, head)	920	11.599	170.425	0	5000
ASF outbreak year (pig offtake per household, head)	920	13.433	180.268	0	5000
One year post-ASF (pig offtake per household, head)	920	6.587	47.257	0	1000
**Information Channels and Sentiment Influence**					
Social media (Importance rank: 1 = most important, 6 = least important)	920	0.801	0.969	0	6
Word of mouth (Importance rank: 1 = most important, 6 = least important)	920	1.065	1.409	0	6
Attention to ASF-related public sentiment on social media (1 = not at all, 5 = very much)	920	3.093	1.263	1	5
Degree of influence of public sentiment on farming decisions (1 = not at all, 5 = very much)	920	2.888	1.401	1	5
**Risk Perception and Behavioral Intentions**					
Plan to raise pigs in the 1–2 years following the ASF outbreak (1 = yes; 0 = no)	920	0.321	0.467	0	1
Reasons for reducing herd size: high epidemic risk (Importance rank: 1 = most important, 6 = least important)	920	0.024	0.227	0	5
Reasons for not raising pigs: high epidemic risk (Importance rank: 1 = most important, 6 = least important)	920	0.509	1.152	0	6

Note: For binary variables, the mean represents the proportion. For the “Reasons” sections, values indicate importance rankings, with 1 = most important and 6 = least important; lower values indicate higher importance. A value of 0 indicates that the respondent did not select the reason. Category headings for groups of indicators are shown in bold.

**Table 4:** Spatial spillover effect of public sentiment on pork market risks.

	Variables	(1) SPDM: Inverse Distance Matrix	(2) SPDM: Gravitational Matrix	(3) SDPD: Inverse Distance Matrix	(4) SDPD: Gravitational Matrix
	Pubsent	0.2688 **	0.2754 **	0.2638 **	0.2702 **
		(0.1096)	(0.1115)	(0.1134)	(0.1153)
	Pig	0.6797 ***	0.6736 ***	0.6805 ***	0.6754 ***
		(0.0247)	(0.0250)	(0.0253)	(0.0256)
	Piglet	−0.0226	−0.0235	−0.0229	−0.0245
		(0.0155)	(0.0155)	(0.0162)	(0.0162)
	Corn	0.0908	0.0928 *	0.0986 *	0.1000 *
		(0.0552)	(0.0561)	(0.0581)	(0.0591)
	Sbm	−0.0741 *	−0.0670 *	−0.0659	−0.0576
		(0.0403)	(0.0399)	(0.0412)	(0.0407)
	Beef	0.0384	0.0186	−0.0167	−0.0519
		(0.1732)	(0.1745)	(0.1805)	(0.1821)
	Mutton	0.1779 *	0.1749	0.1685	0.1674
		(0.1048)	(0.1066)	(0.1074)	(0.1092)
	Broiler	−0.1109 *	−0.1232 *	−0.1180 *	−0.1329 **
		(0.0641)	(0.0647)	(0.0659)	(0.0665)
	Income	0.8724 **	1.0360 **	0.7891 *	0.9378 **
		(0.4260)	(0.4214)	(0.4376)	(0.4350)
	Supply	−0.0017 **	−0.0018 **	−0.0015 *	−0.0017 *
		(0.0008)	(0.0008)	(0.0009)	(0.0009)
	L.WPork			0.0354 *	0.0373 *
				(0.0214)	(0.0212)
	W × Pubsent	0.4042 ***	0.4046 ***	0.3319 **	0.3388 **
		(0.1417)	(0.1414)	(0.1498)	(0.1496)
	W × Pig	−0.2207 ***	−0.1857 ***	−0.2049 ***	−0.1696 ***
		(0.0399)	(0.0390)	(0.0415)	(0.0406)
	W × Piglet	0.0046	0.0117	0.0127	0.0208
		(0.0179)	(0.0173)	(0.0195)	(0.0189)
	W × Corn	0.2626 ***	0.2293 ***	0.2689 ***	0.2313 ***
		(0.0856)	(0.0813)	(0.0894)	(0.0844)
	W × Sbm	−0.0239	−0.0387	−0.0010	−0.0191
		(0.0455)	(0.0435)	(0.0470)	(0.0448)
	W × Beef	−0.1626	−0.1594	−0.2203	−0.1953
		(0.2748)	(0.2541)	(0.2858)	(0.2640)
	W × Mutton	−0.0171	−0.0045	−0.1026	−0.0805
		(0.1507)	(0.1453)	(0.1561)	(0.1502)
	W × Broiler	−0.0393	−0.0153	−0.0387	−0.0137
		(0.1171)	(0.1066)	(0.1205)	(0.1098)
	W × Income	0.3277	−0.1005	0.1978	−0.2170
		(0.5863)	(0.5377)	(0.6284)	(0.5737)
	W × Supply	0.0000	−0.0003	0.0003	−0.0001
		(0.0013)	(0.0013)	(0.0014)	(0.0014)
	ρ	0.3748 ***	0.3325 ***	0.3603 ***	0.3170 ***
		(0.0408)	(0.0397)	(0.0422)	(0.0411)
Direct Effect	Pubsent	0.3441 ***	0.3442 ***	0.3267 ***	0.3283 ***
		(0.1131)	(0.1141)	(0.1108)	(0.1111)
Indirect Effect	Pubsent	0.6222 ***	0.5735 ***	0.5687 ***	0.5309 ***
		(0.1566)	(0.1461)	(0.1890)	(0.1763)
Total Effect	Pubsent	0.9663 ***	0.9177 ***	0.8954 ***	0.8592 ***
		(0.2050)	(0.1914)	(0.2339)	(0.2173)
	N	540	540	510	510
	R^2^	0.8361	0.8398	0.8352	0.8388

Note: Standard errors are shown in parentheses. ***, **, and * indicate statistically significant differences at the 1%, 5%, and 10% levels, respectively.

**Table 5:** Variation in the spatial spillover effect with geographical distance.

Distance Threshold (km)	Spatial Spillover Effect Coefficient	Distance Threshold (km)	Spatial Spillover Effect Coefficient
100	0.0177 ***	1500	0.3576 **
200	0.0855 ***	1600	0.1889 *
300	0.0241 ***	1700	0.1885
400	0.2530 ***	1800	0.2658
500	0.4830 ***	1900	0.2430
600	0.2930 ***	2000	0.1846
700	0.2771 ***	2100	0.1625
800	0.4073 ***	2200	0.2486
900	0.4125 **	2300	0.0645
1000	0.4900 ***	2400	0.0894
1100	0.5548 ***	2500	0.0868
1200	0.4193 **	2600	0.0543
1300	0.2902 **	2700	0.0417
1400	0.5468 **	2800	0.0172

Note: ***, **, and * indicate statistically significant differences at the 1%, 5%, and 10% levels, respectively.

## Data Availability

The original contributions presented in this study are included in the article. Further inquiries can be directed to the corresponding author.
